# Drug repurposing reveals posaconazole as a CYP11A1 inhibitor enhancing anti-tumor immunity

**DOI:** 10.1016/j.isci.2025.112488

**Published:** 2025-04-18

**Authors:** Jhuma Pramanik, Sanu Korumadathil Shaji, Megan Zaman, Bethany Brown, Baojie Zhang, Yumi Yamashita-Kanemaru, Natalie Z.M. Homer, Hosni A.M. Hussein, Qiuchen Zhao, Klaus Okkenhaug, Rahul Roychoudhuri, Abhik Mukhopadhyay, Bidesh Mahata

**Affiliations:** 1 Department of Pathology, University of Cambridge, Cambridge CB2 1QP, UK; 2 Mass Spectrometry Core, Edinburgh Clinical Research Facility, Centre for Cardiovascular Science, Queen’s Medical Research Institute, University of Edinburgh, Edinburgh, UK; 3 Cambridge Crystallographic Data Centre, Cambridge CB2 1EZ, UK

**Keywords:** Pharmacology, Natural sciences, Biological sciences, Systems biology, Cancer systems biology

## Abstract

Steroid hormones regulate cell physiology and immune function, with dysregulated steroidogenesis promoting cancer progression by supporting tumor growth and suppressing anti-tumor immunity. Targeting CYP11A1, the first and rate-limiting enzyme in steroid biosynthesis, has shown promise in cancer therapy, but safe and effective inhibitors remain an unmet need. Undertaking *in silico* structure-based drug repurposing approach, we found posaconazole as an inhibitor of CYP11A1. The docking pose analysis showed that posaconazole can form multiple hydrogen bonds and hydrophobic interactions with the key residues at the binding site and the cofactor, stabilizing the protein-ligand complex. We validated its inhibition efficiency in cell-based assays. In a mouse model of lung metastasis, we demonstrated that posaconazole restricts metastasis by stimulating anti-tumor immunity. These findings highlight posaconazole’s potential as a research tool to study steroidogenesis and as a candidate for further preclinical and clinical evaluation in pathologies associated with local steroidogenesis, such as steroidogenic tumors.

## Highlights

• Drug repurposing identifies posaconazole as an inhibitor of CYP11A1
• Posaconazole inhibits steroidogenesis
• Posaconazole induces anti-tumor immunity and restricts lung metastasis in mice

## Graphical abstract

**Figure:**
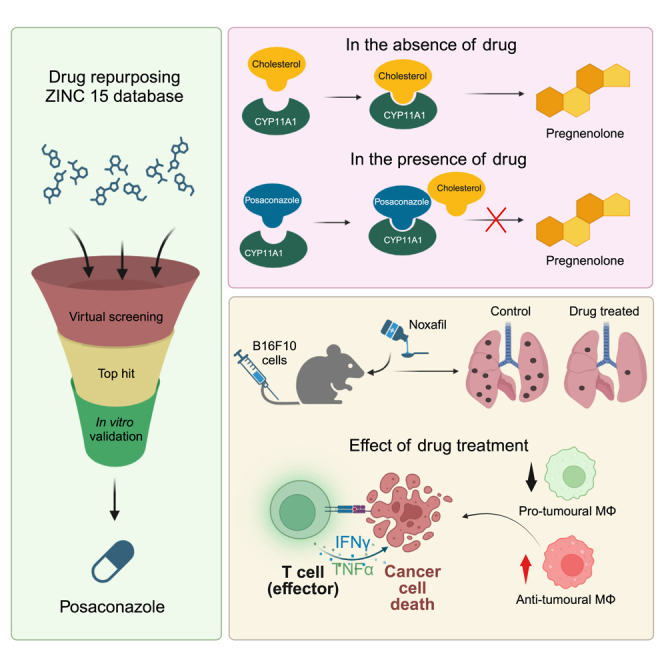

## Introduction

CYP11A1, also known as P450 side chain cleavage enzyme or P450scc, is a crucial member of the cytochrome P450 family. This enzyme catalyzes the initial and rate-limiting step in steroidogenesis by converting cholesterol to pregnenolone.^1^^,^^2^ Pregnenolone serves as the first bioactive steroid in the steroid biosynthesis pathway and acts as a precursor for all other steroid hormones.^3^ The conversion of cholesterol to other steroid hormones is termed as steroidogenesis, and sometimes this has been denoted as *de novo* steroidogenesis to distinguish it from the local conversion of steroids from one species to another.^4^ CYP11A1 is present in the inner mitochondrial membranes of steroidogenic cells.^1^^,^^2^ Steroid-producing cells are commonly found in the adrenal gland, gonads, and placenta, which are the major contributors to the systemic level of steroids in the body.^1^^,^^2^^,^^5^ However, extra-glandular steroidogenesis, also known as local steroidogenesis, is reported in various tissues such as the brain,^1^^,^^6^ skin,^7^ thymus,^8^ lung,^9^ intestine,^10^^,^^11^ and adipose tissues.^12^ Interestingly, several types of immune cells have also been identified to produce steroids.^4^^,^^13^^,^^14^^,^^15^^,^^16^^,^^17^

Steroid hormones play crucial roles in diverse physiological processes, including cellular metabolism regulation, electrolyte balance, reproductive organ differentiation, pregnancy maintenance, and immune cell function modulation.^4^ The immunosuppressive properties of steroids have been extensively investigated and utilized clinically for treating various immune-mediated disorders.^18^ Recent studies have demonstrated that the presence of steroids in the tumor microenvironment (TME) promotes cancer progression and metastasis by suppressing anti-tumor immune responses.^13^^,^^15^^,^^19^^,^^20^ Notably, steroid production by immune cells within the TME contributes to the remodeling of the local environment, leading to suppressed immune surveillance and facilitating cancer cell immune evasion.^13^^,^^15^ Understanding the fundamental mechanisms of tumor-induced immunosuppression within the TME is critical for developing novel therapeutic strategies in cancer immunotherapy.^21^ Genetic and pharmacological studies targeting CYP11A1, a key enzyme in steroidogenesis, have suggested the potential of modulating this pathway to reinvigorate anti-tumor immunity.^10^^,^^12^^,^^16^ A substantial body of experimental evidence supports the occurrence of local (extra-adrenal) steroid production within tumors, either through *de novo* steroidogenesis or steroid conversion. This phenomenon has been observed in various malignancies, including cutaneous, gastrointestinal, prostate, and bone metastatic cancers, among others.^7^^,^^10^^,^^20^^,^^22^^,^^23^^,^^24^ Beyond oncology, elevated steroidogenesis has been implicated in other pathological conditions, such as peanut allergy, where CYP11A1 inhibition has been proposed as a potential therapeutic approach.^17^^,^^25^ However, the development of a safe and efficacious CYP11A1 inhibitor remains an unmet clinical need. The identification of a potent steroidogenic inhibitor could have wide-ranging applications, as small molecule inhibition of CYP11A1 could effectively target the *de novo* steroid synthesis pathway in various pathological conditions. Given the multifaceted potential of CYP11A1 inhibition, this study aims to identify a novel CYP11A1 inhibitor that can serve both as a valuable research tool for investigating steroidogenesis and as a promising candidate for future therapeutic development.

In this study, we employed a drug repurposing strategy to identify novel CYP11A1 inhibitors, leveraging the efficiency and cost-effectiveness of structure based virtual screening. We utilized the GOLD docking suite to screen three subsets of compounds from the ZINC15 database: FDA-approved drugs, drugs in global use, and compounds under clinical investigation. This approach aimed to overcome the time delays, high attrition rates, and substantial resource commitments associated with traditional drug discovery methods. Our screening process identified posaconazole, an FDA-approved antifungal drug, as a potent inhibitor of CYP11A1 activity. *In vitro* studies demonstrated that posaconazole effectively attenuates pregnenolone synthesis in *de novo* steroidogenic T helper 2 (Th2) cells, outperforming aminoglutethimide, the current gold standard inhibitor of steroidogenesis. To evaluate the *in vivo* efficacy of posaconazole, we employed a mouse lung metastasis model using B16-F10 metastatic melanoma cells. Our results showed that posaconazole treatment significantly inhibited tumor progression by reinstating anti-tumor immunity. Specifically, posaconazole increased the number of anti-tumoral T cells and M1 macrophages while reducing pro-tumoral M2 macrophages in the TME. These findings highlight the potential of repurposing posaconazole for the therapeutic inhibition of steroidogenesis in pathological conditions associated with elevated steroid production. Moreover, our study provides a valuable tool for biological research, offering a means to inhibit the first and rate-limiting step of the steroid biosynthesis pathway. This advancement will enhance our analytical understanding of *de novo* steroidogenesis and facilitate further investigations into steroid-dependent processes in various physiological and pathological contexts.

## Results

### Virtual screening and selection of compounds for *in vitro* evaluation

**Figure 1:**
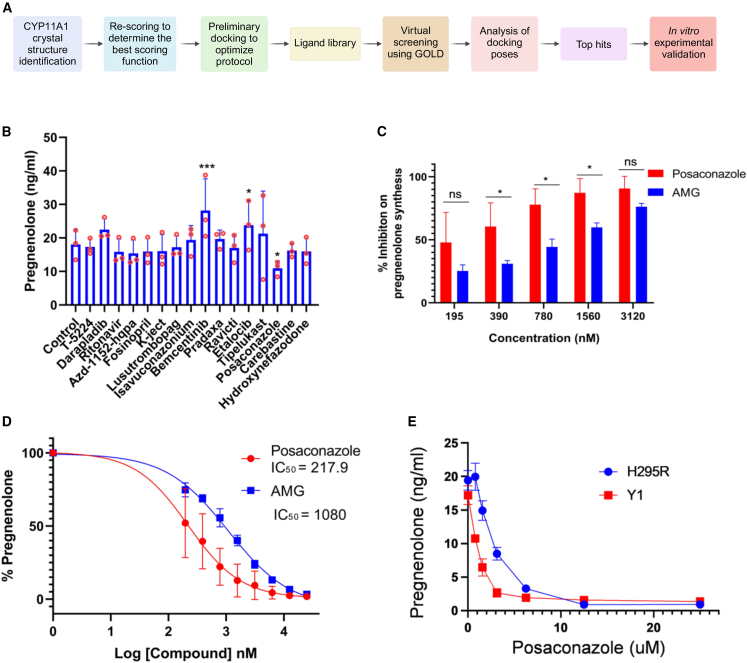
*In vitro* validation of top hits and identification of posaconazole as a potent inhibitor of CYP11A1 (A) Schematic of the pipeline used for the identification and validation of CYP11A1 inhibitors using *in silico* drug repurposing approach. (B) Quantification of pregnenolone synthesis by Th2 cells after treatment with 1 μM of shortlisted drugs. *In vitro* generated Th2 cells were cultured in the presence of different drugs for 48 h in charcoal stripped serum containing media. Pregnenolone concentration was measured by ELISA (*N* = 3, *N* represents independent biological repeats with Th2 cells derived from three individual mice. One-way ANOVA with Fisher’s LSD test; ∗*p* ≤ 0.05, ∗∗*p* ≤ 0.01, ∗∗∗*p* ≤ 0.001). Error bars represent mean with SD. (C) Dose dependent inhibition of pregnenolone synthesis in the presence of posaconazole and aminoglutethimide. *In vitro* generated Th2 cells were treated with different concentration of posaconazole and aminoglutethimide for 48 h and pregnenolone concentration was measured by ELISA (*N* = 3, *N* represents independent biological repeats with Th2 cells derived from three individual mice. Two-way ANOVA with Sidak multiple comparison; ∗*p* ≤ 0.05, ∗∗*p* ≤ 0.01, ∗∗∗*p* ≤ 0.001). Error bars represent mean with SD. (D) IC_50_ calculation of CYP11A1 inhibition by posaconazole and aminoglutethimide. Error bars represent mean with SD*. N* = 3, *N* represents independent biological repeats. (E) H295R and Y1 cells were culture in the presence of increasing concentration of posaconazole and pregnenolone concentration in the cell culture supernatant was measured by quantitative ELISA. Error bars represent mean with SEM*. N* = 3, *N* represents three independent repeats with the same cell line in different experimental conditions. Representative result of three independent experiments with similar results.

A brief overview of the screening methodology is represented in Figure 1A. Four structures of CYP11A1 from human are available in The Protein DataBank in Europe (PDBe) with IDs 3N9Y, 3NA0, 3NA1, and 3N9Z.^26^ The resolution, R factor, and R_free_ values for all these structures were compared (Table S1). All the structures were compared for clashes or other potential model building errors within the active site residues. 3N9Y structure had clashes at S352, V353, and I461, whereas 3NA1 had a sidechain outlier at S352 and a class with the Q377 residue indicating model building errors near the active site. 3NA0 possessed more fit-to-density (RSRZ) and Ramachandran outliers. 3N9Z was selected and used for all docking experiments. 3N9Z has 22-hydroxycholesterol (22-HC) (PDB chem comp id HC9) as the co-crystallized ligand. Analysis of binding pockets, using SuperStar, of 3N9Z showed both hydrophilic and hydrophobic regions (Figures S1A and S1B). Aliphatic carbon was used to probe the hydrophobic regions, whereas alcohol carbon was used to find hydrophilic regions.

After virtual screening of the 7,190 compounds from the aforementioned libraries from ZINC-15, a total of 19 compounds (Figure S1C) were shortlisted for further experimental analysis. In this final list, we have carefully included compounds that can form polar and non-polar interactions based on the SuperStar analysis of CYP11A1 binding pockets, where both hydrophilic and hydrophobic regions were observed (Figures S1A and S1B).

### *In vitro* validation of shortlisted CYP11A1 inhibitors identifies posaconazole as a potent inhibitor of steroidogenesis

Th2 cells express Cyp11a1, which converts cholesterol to pregnenolone.^13^^,^^14^ However, they do not express downstream enzymes that can catalyze pregnenolone to other steroids.^13^ Therefore, Th2 lymphocytes are ideal to test Cyp11a1 inhibition by measuring pregnenolone concentration. To shortlist the drugs with any Cyp11a1 inhibitory effect, *ex vivo* differentiated murine Th2 cells were treated with 10 μM drugs for 48 h, and the inhibitory effect was measured by pregnenolone ELISA (Figure S1D). Next, to compare the inhibitory efficiency of the selected drugs with aminoglutethimide, 1 μM of the shortlisted compounds were added to the Th2 culture for 48 h and pregnenolone levels were quantified (Figure 1B). The dose was selected based on preliminary titration experiments in this study where aminoglutethimide demonstrated Th2 steroidogenesis inhibition at sub-micromolar concentrations (Figure 1C). The ELISA analysis identified strong inhibition of pregnenolone synthesis in Th2 cells treated with posaconazole (Figure 1B). The drugs oprozomib, octenidine, and itraconazole showed 94.12%, 57.55%, and 86.58% cytotoxicity in Th2 cells, respectively (Figure S1D). Therefore, these compounds were excluded from further downstream analysis. Posaconazole inhibited the pregnenolone synthesis without any significant reduction in cell viability (Figure S1E). This result indicated the successful identification of a CYP11A1 inhibitor using the drug repurposing pipeline established to perform the current study. Further *in vitro* and *in silico* analyses were performed on posaconazole to characterize its potential to inhibit CYP11A1 activity. Since posaconazole showed a reduction in pregnenolone synthesis without significant change in the cell viability, a dose-dependent analysis of posaconazole on CYP11A1 inhibition was performed using pregnenolone ELISA. Th2 cells were treated with posaconazole and aminoglutethimide with concentrations ranging from 195–3,120 nM for 48 h. The analysis showed a dose-dependent reduction in pregnenolone levels, indicating an inhibition in CYP11A1 activity (Figure 1C). The IC_50_ value for the posaconazole was found to be 217.9 nM, which was four times lower than that of aminoglutethimide, which showed an IC_50_ of 1,080 nM (Figure 1D). Aminoglutethimide is the gold-standard CYP11A1 inhibitor and widely used in research. The GOLD score of aminoglutethimide obtained in the virtual screening was 60, whereas posaconazole scored 112.4 (Table S2), which is also in line with the inhibitory activity observed *in vitro*. These observations indicate that posaconazole is a potent inhibitor of CYP11A1 activity that outperforms the well-established CYP11A1 inhibitor aminoglutethimide in preventing pregnenolone synthesis in Th2 cells.

### Multiple interactions stabilize posaconazole in CYP11A1 binding pocket

**Figure 2:**
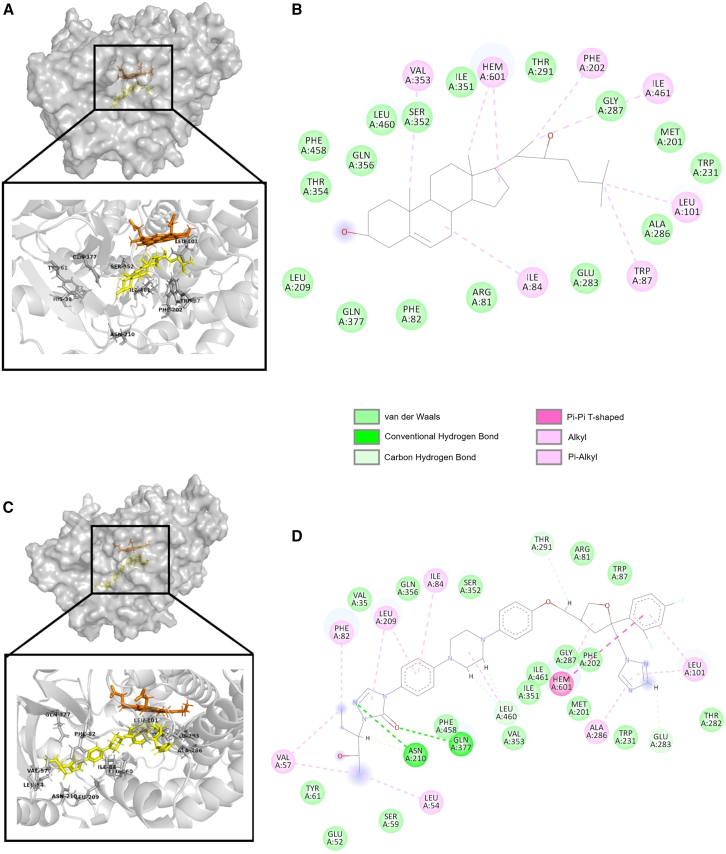
CYP11A1 crystal structure (PDB ID: 3N9Z) and docking pose of posaconazole generated by GOLD suite (A) Surface structure of the CYP11A1 protein (cartoon, gray) with 22-hydroxycholesterol (stick, yellow). Inset shows the co-crystallized ligand and the key residues involved in the ligand protein interactions. Heme co-factor is shown as stick in orange. (B) 2D interaction diagram of 22-hydroxycholestrol created using discovery studio visualizer. (C) Surface structure of posaconazole CYP11A1 complex. Inset shows focused image of ligand binding pocket of CYP11A1 (cartoon, gray) with posaconazole (stick, yellow) and heme cofactor (stick, orange). The interacting residues in the protein are labeled and represented as gray sticks. (D) 2D diagram of ligand and protein interaction created using discovery studio visualizer. Residues forming various interactions with posaconazole (hydrogen bonds, carbon hydrogen bonds, van der Waals interaction, pi-alkyl interaction, pi-pi interactions, and alkyl interactions) are denoted in the diagram.

Posaconazole’s CYP11A1 inhibitory potential observed in the *in vitro* experiments can be explained by its interactions in the docking pose obtained in the virtual screening (Figure 2). The ligand-protein complex is stabilized by multiple hydrogen bonds and hydrophobic interaction. The bulky nature of posaconazole fits very well in the large binding pocket of CYPA11A1. Two strong hydrogen bond interactions were observed in the complex in which the nitrogen in the azole group forms the first hydrogen bond with Asn210 residue, whereas the oxygen atom in the same azole group forms the second hydrogen bond with Gln377 residue (Figures 2C and 2D). *In silico* mutagenesis of these two key residues, Asn210 and Gln377, to alanine followed by docking showed that Gln377 contributed more to the stability of the posaconazole-CYP11A1 complex (Figures S2A–S2C) and resulted in elimination of hydrogen bonding interactions with both Asn210 and Gln377. Mutation of Gln377 to alanine residue has resulted in elimination of the key hydrogen bonding interaction between Gln377 and posaconazole. The distance between azole oxygen of posaconazole and Gln377Ala has increased from 3 Å to 6.7 Å in the docking pose after mutation and resulted in a less stable docking pose indicated by docking score (Figure S2B). However, after mutating to alanine, in the absence of Asn210, posaconazole could retain the hydrogen bonding interaction with Gln377. Posaconazole has an N-aryl piperazine backbone attached to the phenyl and azole group suitable for hydrophobic interactions. Non-polar interactions stabilizing the posaconazole-CYP11A1 complex is contributed by amino acids such as Glu52, Tyr61, Ser59, Val57, Leu54, Phe82, Val35, Iue209, Gln356, Ile84, Ser352, Arg81, Trp87, Leu101, Thr282, Trp231, Ala286, Met201, Phe202, Gly287, Ile461, Ile351, Val353, and Phe458. Among the non-polar interactions, the terminal 2,4-difluorophenyl in the posaconazole forms a pi-pi (π/π) T-shaped interaction with the Heme group, which further stabilizes the complex. Val57, Ph82, Leu209, Ile84, Leu101, Ala286, Leu460, and Leu54 form alkyl and π-alkyl interaction between posaconazole and CYP11A1. Stabilization of ligand structure in the active by van der Waals interactions was orchestrated by amino acid residues such as Glu52, Tyr61, Ser59, Val35, Ser352, Arg81, Trp87, Thr282, Trp231, Met201, Phe202, Gly287, Ile461, Ile351, Val353, and Phe458. These interactions facilitate the formation of a strong protein-ligand complex, which prevents cholesterol from accessing the CYP11A1 active site restricting the formation of pregnenolone, thereby forestalling the steroid biosynthesis pathway.

### Oral administration of posaconazole restricts experimental lung metastasis by enhancing anti-tumor immunity

**Figure 3:**
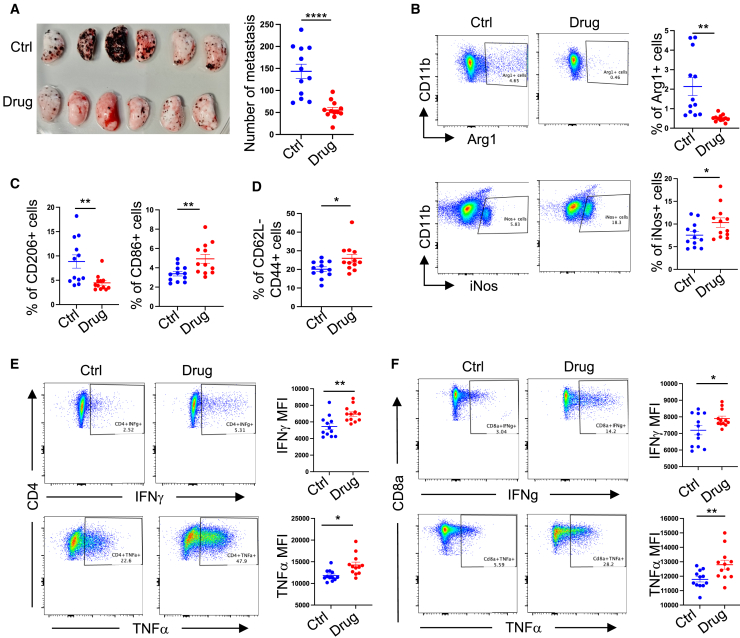
Effect of oral administration of posaconazole in lung colonization of B16F10 melanoma cells B16F10 cells were injected in C57BL/6 mice in the tail vein. A globally approved formulation of posaconazole, Noxafil 40 mg/mL oral suspension, was administered to the experimental mice of metastatic melanoma by oral gavage at a dose of 20 mg/kg body weight, daily, for 10 days. Lungs were harvested after 12 days. (A) Left: representative photograph of pulmonary metastatic foci produced 12 days after tail vein injection of B16-F10 cells in posaconazole-treated and control (drug and ctrl, respectively) mice. Left lobes of the lungs are shown. *N* = 11–12. Right: graphical presentation of the numbers of lung metastatic foci. Each point represents an individual animal, error bars represent mean with SEM. Unpaired two-tailed t test*.* (B–D) Comparing immunophenotype in posaconazole (drug) treated and vehicle-treated (ctrl) mice with B16-F10 cells injected intravenously. *N* = 11–12. (B) Representative fluorescence-activated cell sorting (FACS) profile and respective graphical presentation of iNOS and Arg1 expression in lung-infiltrating live, singlet, CD45^+^Lin^−^ CD11b^+^ F4/80^+^ macrophages. (C) Respective graphical presentation of CD206 and CD86 expression in lung-infiltrating live, singlet, CD45^+^Lin^−^ CD11b^+^ F4/80^+^ macrophages. (D) Representative graphical presentation of CD62L^−^ CD44^+^ CD8 T cells. (E and F) Representative FACS profile and respective graphical presentation of IFN-γ and TNF-α expression in tumor infiltrating CD4 (E) and CD8 T (F) cells. FACS gating: all cells > singlets > live cells > CD45^+^ > (B) Lin^−^CD11b^+^F4/80^+^ > CD11b^+^iNOS^+^ or CD11b^+^Arg1^+^ (C) Lin^−^ CD11b^+^ F4/80^+^ > CD206 or CD86 (D) Lin^−^ TCRβ^+^ > CD8^+^ >CD63L^−^ CD44^+^ (E and F). Lin^−^ TCRβ^+^ > CD4^+^ or CD8^+^ > IFN-γ or TNF-α. In this figure, all error bars represent mean with SEM. *p* value was calculated by unpaired two-tailed t test. ∗*p* ≤ 0.05, ∗∗*p* ≤ 0.01, ∗∗∗*p* ≤ 0.001. *N* represents biologically independent individual animals.

Previously, it has been shown that genetic deletion or pharmacologic inhibition of Cyp11a1 improves anti-tumor immunity and restricts experimental tumor growth.^13^ Therefore, to test the effect of posaconazole *in vivo*, we administered a globally approved formulation of posaconazole, Noxafil 40 mg/mL oral suspension, to the experimental mice of metastatic melanoma at a dose of 20 mg/kg body weight, daily, for 10 days. On 12^th^ day, lungs were harvested to analyze the tumor burden and immune cell phenotype. We observed a significant reduction in metastatic colonies in the posaconazole-treated mice compared to the vehicle-treated mice (Figure 3A). Immunophenotyping of the lung was performed using flow cytometry. We found that posaconazole treatment increased the number of iNOS^+^CD11b1^+^F4/80^+^ cells and reduced the number of Arg1^+^CD11b1^+^F4/80^+^ cells in the lung (Figure 3B). Increased iNOS expression in the macrophage suggests the increased M1 macrophages, which are known as anti-tumoral. By contrast, decreased Arg1 expression in the macrophage population suggests the decreased M2 macrophages, which are known as immunosuppressive and pro-tumoral. The observations of changes in M1:M2 because of drug treatment was further validated by decreased CD206 expression and increased CD86 expression (Figure 3C). In posaconazole-treated mice, CD62L^−^CD44^+^ activated T cells were increased in frequency (Figure 3D). Both CD4^+^ and CD8^+^ T cells express more interferon (IFN)-γ and tumor necrosis factor alpha (TNF-α) in drug treated mice compared to vehicle-treated mice (Figures 3E and 3F). We observed no significant change in the systemic level of steroids as revealed by steroid profiling of control and posaconazole treated mice serum (Figure S3).

These results collectively demonstrate that oral administration of posaconazole effectively restricts experimental lung metastasis by reinvigorating anti-tumor immunity *in vivo*. The observed changes in immune cell composition and function suggest a shift toward a more favorable, tumor-suppressive microenvironment. This study provides compelling evidence for the potential of posaconazole as an immunomodulatory agent in cancer therapy, warranting further investigation into its clinical applications.

## Discussion

Immunosuppression and cancer immune evasion is a therapeutic challenge in developing effective immunotherapy.^21^ Based on mice model-based experimental evidence, it has been proposed that targeting CYP11A1 may reinstate anti-tumor immunity.^13^^,^^15^ Therefore, in this study, we aimed to identify a new inhibitor of CYP11A1 and test its anti-tumor efficacy.

The study successfully employed a drug repurposing approach to identify posaconazole as a potent inhibitor of CYP11A1, outperforming the established inhibitor aminoglutethimide. The in silico structure based virtual screening of 7,190 compounds from the ZINC15 database, followed by rigorous *in vitro* validation, led to this discovery. This approach not only accelerates the drug discovery process but also leverages existing safety and pharmacokinetic data, potentially expediting clinical translation.

The superior inhibitory effect of posaconazole can be attributed to its molecular structure and interactions within the CYP11A1 binding pocket. Posaconazole’s larger size (700.8 g/mol) compared to aminoglutethimide (232.28 g/mol) allows for better occupancy of the CYP11A1 binding cavity. Unlike posaconazole, aminoglutethimide does not display any hydrogen bonding interactions with any of the key residues present in the active site. The hydrophobic ring structures of posaconazole align well with the hydrophobic regions of the active site, mimicking the natural ligand cholesterol. In the docked conformation of CYP11A1-posaconazole complex, the terminal phenyl-2-hydroxypentan-triazolone group is positioned near to the surface away from the heme group. The presence of this group is critical for the stability of the complex by forming polar interactions in the active site. Posaconazole forms two critical hydrogen bonds: one between its nitrogen atom and Asn210, and another between its oxygen atom and Gln377, present in the triazolone group. In the docked conformation of CYP11A1-posaconazole complex, the terminal phenyl-2-hydroxypentan-triazolone group is positioned near to the surface away from the heme group. The presence of this group is critical for the stability of the complex by forming polar interactions in the active site. Leu101 forms alkyl interactions with the 1,2,4 triazole group and p-alkyl interactions with the 2,4-difluorophenyl ring. The 2,4-difluorophenyl ring of posaconazole engages in pi-pi interactions with the heme cofactor. Another important residue involved in positioning native ligand cholesterol’s C22 atom near to the heme cofactor is Ser352, along with the hydrogen-bonded water molecule.^26^ The Ser352 residue forms van der Waals interaction to stabilize the protein-ligand complex in the posaconazole docked conformation. These interactions collectively stabilize the posaconazole-CYP11A1 complex, effectively blocking cholesterol access to the active site and inhibiting pregnenolone synthesis.

The dose-dependent inhibition of pregnenolone synthesis by posaconazole suggests its potential application in cancer immunotherapy. Our *in vivo* studies demonstrated that oral administration of posaconazole restricts tumor growth in a mouse model of metastatic melanoma by reinvigorating anti-tumor immunity. Posaconazole treatment increased anti-tumoral M1 macrophages and decreased pro-tumoral M2 macrophages in the lung. The drug increased the frequency of activated T cells and enhanced IFN-γ and TNF-α expression. These findings suggest that posaconazole can reshape the TME to favor anti-tumor immune responses.

Posaconazole provides an immediate benefit as a research tool to study steroidogenesis, offering a means to inhibit the first and rate-limiting step of the steroid biosynthesis pathway. Beyond cancer, posaconazole’s CYP11A1 inhibition could be explored in other conditions characterized by elevated steroidogenesis, such as peanut allergy. As an FDA-approved antifungal medication, posaconazole’s safety profile and pharmacokinetics are well established, potentially accelerating its translation to new therapeutic applications. The study provides valuable insights into the structural basis of CYP11A1 inhibition, which could guide future drug design efforts targeting this enzyme.

### Limitations of the study

While our study demonstrates the potential of posaconazole as a CYP11A1 inhibitor, several limitations present opportunities for further research. Expanding the compound library beyond approved drugs could potentially identify even more potent CYP11A1 inhibitors. Structural biology investigations, such as co-crystallization of posaconazole with CYP11A1, along with introduction of point mutations in the key CYP11A1-posaconazole interacting sites would provide valuable insights into their precise interactions. *In vitro* studies involving mutations of the predicted interacting residues could further validate our in silico observations. The molecular mechanisms underlying posaconazole’s immunomodulatory effects require further elucidation. Potential off-target effects of posaconazole were not extensively explored in this study. An *in vivo* comparative study with aminoglutethimide, approved immunotherapies, such as checkpoint inhibitors, or other cancer drugs would be an important future direction. These limitations provide opportunities for future research to fully understand posaconazole’s potential as a cancer immunotherapy agent, underscoring the promising foundation laid by our study for exploring its role in immunomodulation and cancer prevention through targeting immune cell-mediated steroidogenesis.

In conclusion, this study identifies posaconazole as a promising CYP11A1 inhibitor with potential applications in cancer immunotherapy and other conditions characterized by elevated steroidogenesis. The immediate benefit lies in its use as a research tool to study steroidogenesis, while its clinical potential warrants further preclinical and clinical investigation.

## Resource availability

### Lead contact

Further information and requests for resources and reagents should be directed to and will be fulfilled by the lead contact, Bidesh Mahata (bm562@cam.ac.uk).

### Material availability

This study did not generate unique reagents.

### Data and code availability

• No large dataset was generated in this study. All data are available in the main article and supporting files, and will be shared by the lead contact upon request.
• This paper does not report original code.
• Any additional information required to reanalyze the data reported in this paper is available from the lead contact upon request.

## Acknowledgments

This study is supported by 10.13039/501100000289CRUK Career Development Fellowship (RCCFEL∖100095), 10.13039/100000001NSF-BIO/10.13039/501100000268UKRI-BBSRC project grant (BB/V006126/1); and 10.13039/501100000265MRC project grant (MR/V028995/1). J.P. is supported by 10.13039/100000001NSF-BIO/10.13039/501100000268UKRI-BBSRC project grant (BB/V006126/1) and 10.13039/501100000265MRC project grant (MR/V028995/1).

## Author contributions

Conceptualization, B.M. and A.M.; methodology, B.M., S.K.S., and J.P.; investigation, J.P. (wet lab, *in vivo*, and *in vitro*), S.K.S. (*in silico* and *in vitro*), M.Z., B.B. (preliminary work), B.Z., Y.Y.-K. (*in vivo*), and N.Z.M.H. (steroid analysis); data curation and visualization, J.P., S.K.S., and N.Z.M.H. (steroid detection); writing – original draft, S.K.S., J.P., and B.M.; writing – review & editing, all authors; supervision, B.M. (overall), R.R. (immunology), K.O. (signaling), N.Z.M.H. (steroid detection), and A.M. (computational); funding acquisition, B.M.; project administration, B.M.

## Declaration of interests

Based on this study, an UK patent application is pending on the use of posaconazole in cancer therapy of breast and metastatic melanoma.

## STAR★Methods

### Key resources table

**Table:** 

REAGENT or RESOURCE	SOURCE	IDENTIFIER
**Antibodies**		

CD3e Monoclonal Antibody (Clone 145-2C11)	eBioscience	Cat#14-0031-82; RRID: AB_468847
CD28 Monoclonal Antibody (Clone 37.51)	eBioscience	Cat#14-0281-82; RRID: AB_467190
Ultra-LEAF™ Purified anti-mouse IFN-γ Antibody (Clone: XMG1.2)	Biolegend	Cat#505834; RRID: AB_2616676
Ultra-LEAF™ Purified anti-mouse IL-12/IL-23 p40 (Clone: C17.8)	Biolegend	Cat# 505308; RRID: AB_11148948
Brilliant Violet 785™ anti-mouse F4/80 Antibody (Clone: BM8)	Biolegend	Cat#123141; RRID: AB_2563667
CD45 Monoclonal Antibody (30-F11), Brilliant Ultra Violet™ 563 (Clone: 30-F11)	eBioscience	Cat#365-0451-82; RRID: AB_2925377
redFluor™ 710 Anti-Human/Mouse CD11b (Clone: M1/70)	Tonbo Biosciences	Cat#80-0112-U100
FITC Anti-Mouse TCR beta (Clone: H57-597)	Tonbo Biosciences	Cat#35-5961-U500
CD19 Monoclonal Antibody, FITC(Clone: eBio1D3 (1D3))	eBioscience	Cat# 11-0193-85; RRID: AB_657668
CD4 Monoclonal Antibody, FITC (Clone: RM4-5)	eBioscience	Cat#11-0042-82; RRID: AB_464896
CD8a Monoclonal Antibody, FITC, (Clone: 53-6.7)	eBioscience	Cat#11-0081-85; RRID: AB_464916
CD86 Monoclonal Antibody, PE-Cyanine7, (Clone: GL1)	Invitrogen	Cat#A15412, RRID: AB_2534425
PerCP/Cyanine5.5 anti-mouse CD206 (MMR) Antibody (Clone: C068C2)	Biolegend	Cat#141716; RRID: AB_2561991
PE/Cyanine7 anti-mouse CD4 Antibody (Clone: GK1.5)	Biolegend	Cat#100422; RRID: AB_312706
CD8a Monoclonal Antibody, APC-eFluor™ 780 (Clone: 53-6.7)	eBioscience	Cat#47-0081-82; RRID: AB_1272185
IFN gamma Monoclonal Antibody, PerCP-Cyanine5.5, (Clone: XMG1.2)	eBioscience	Cat#45-7311-82; RRID: AB_1107020
TNF alpha Monoclonal Antibody, PE-eFluor™ 610, (Clone: MP6-XT22)	eBioscience	Cat# 61-7321-82; RRID: AB_2574666
iNOS Monoclonal Antibody, APC, (Clone: CXNFT)	eBioscience	Cat# 17-5920-82; RRID: AB_2573244
Arginase 1 Monoclonal Antibody, PE-Cyanine7, (Clone: A1exF5)	eBioscience	Cat# 25-3697-82; RRID: AB_2734841

**Chemicals, peptides, and recombinant proteins**		

Recombinant Mouse IL-2 Protein	R&D Systems	Cat# 402-ML
Recombinant Mouse IL-4 Protein	R&D Systems	Cat# 404-ML
Collagenase D	Roche	Cat#11088858001
Collagenase A	Roche	Cat#10103578001
DNase I	Roche	Cat#10104159001
Monensin	Biolegend	Cat#420701
IC fixation buffer	eBioscience	Cat#00-8222-49
β-mercaptoethanol	Gibco	Cat#21985023
Charcoal-stripped Fetal Bovine Serum	Sigma-Aldrich	Cat#F6765
Iscove’s Modified Dulbecco’s Medium	Fisher Scientific	Cat# 11500556
Phorbol 12-myristate 13-acetate (PMA)	Sigma-Aldrich	Cat#P1585
Ionomycin calcium salt	Sigma-Aldrich	Cat#I0634

**Critical commercial assays**		

Pregnenolone ELISA Kit	Abnova	Cat# KA1912
Acridine Orange (AO) and DAPI Staining Reagent	Chemometec	Cat# 910-3018
NucleoCounter NC-250 automated cell counter	Chemometec	Cat# 970-0251
CD4^+^ T Cell Isolation kit	Miltenyi Biotec	Cat#130-104-454
LIVE/DEAD™ Fixable Violet Dead Cell Stain Kit, for 405 nm excitation	Invitrogen	Cat#L34964

**Deposited data**		

Clustering of top 100 compounds	This paper	Table S2

**Experimental models: Cell lines**		

B16-F10	ATCC	CRL-6475
H295R	ATCC	CRL-2128
Y1	ATCC	CCL-79

**Software and algorithms**		

ZINC15 database	Sterling et al.^27^	https://zinc15.docking.org
KNIME	KNIME Inc.	https://www.knime.com
RDKit		https://www.rdkit.org
CCDC GOLD suite	Jones et al.^28^	https://www.ccdc.cam.ac.uk/solutions/software/gold
DataWarrior	Sander et al.^29^	https://openmolecules.org/datawarrior
PDBe	Armstrong et al.^30^	https://www.ebi.ac.uk/pdbe
CCDC Mogul	Cottrell et al.^31^, Bruno et al.^32^	https://www.ccdc.cam.ac.uk/solutions/software/mogul
GraphPad Prism	*GraphPad*	https://www.graphpad.com/updates/prism-843-release-notes
BioRender	BioRender	https://app.biorender.com

### Experimental model and study participant details

#### Mice

In this investigation, all mice were handled and cared for following the stringent guidelines set forth by the UK Animals in Science Regulation Unit’s Code of Practice for the Housing and Care of Animals Bred, Supplied, or Used for Scientific Purposes, and the Animals (Scientific Procedures) Act 1986 Amendment Regulations 2012. All experimental protocols were conducted under the authorization of a UK Home Office Project License (PPL P0AB4361E) and received the necessary approval from the local institute’s Animal Welfare and Ethical Review Body, ensuring compliance with ethical standards and animal welfare considerations.

The sample size was determined according to our previous experience and *a priori* power analysis (G∗Power). Housing condition of Gurdon animal facility: all mice used in this study were maintained in specific pathogen free unit on 12 hours light and 12 hours dark cycle. The mice were genotyped by Transnetyx. In this study we used both male and female mice aged between 8 to 12 weeks.

### Method details

#### B16-F10 melanoma metastasis model and posaconazole treatment

B16-F10 melanoma cell line was purchased from American Type Culture Collection (ATCC) and cultured in Dulbecco’s Modified Eagle medium (DMEM, Life Technologies), supplemented with 100 U/mL penicillin/streptomycin and 10% foetal bovine serum (FBS; Life Technologies, Invitrogen). For metastasis, 5 × 10^5^ B16-F10 cells in a volume of 0.1 ml PBS were injected intravenously into the tail vein of wild-type (WT) C57BL/6 mice. Mice received 200μL posaconazole (Noxafil, 40 mg/ml oral suspension) by oral gavage at 20 mg/kg body weight, diluted in water, once daily.^33^^,^^34^^,^^35^ The first dose was administered concurrently with cell injection and continued for 10 days. The vehicles were administered orally by gavage in control mice. After 12 days, animals were killed and perfused the lung with 10 ml PBS, and tissues were collected for analysis. The number of B16-F10 colonies on all five lobes of the lung was counted macroscopically.

#### Steroid profiling of mice serum

Multiple steroids were measured in serum samples by targeted liquid chromatography tandem mass spectrometry (LC-MS/MS).^36^ Briefly, serum was enriched with a mixture of isotopically labelled steroids as internal standards and steroids were extracted by supported liquid extraction on an Extrahera liquid handling robot by Biotage (Uppsala, Sweden). The extract was subject to chromatographic separation on an Acquity I-Class UPLC by Waters (Wilmslow, UK) fitted with a Kinetex C18 (150 × 2.1 mm; 2.6 μm) chromatography column by Phenomenex (Macclesfield, UK). Steroids were separated using a 16-minute gradient mobile phase consisting of water and methanol, both with 0.05 mM ammonium fluoride, with a flow rate of 0.3 mL/min and a column temperature of 50C. Separated steroids were analysed on a QTrap 6500+ by AB Sciex (UK) using multiple reaction monitoring in both positive and negative ion mode. Concentrations of steroid in the sample were calculated using MultiQuant software by AB Sciex (Macclesfield, UK).^37^

#### Lung tissue processing for metastatic melanoma analysis

The lungs from metastatic melanoma bearing mice were mechanically dissociated and were then subjected to enzymatic digestion using a mixture of 1 mg/ml collagenase D (Roche), 1 mg/ml collagenase A (Roche), and 0.4 mg/ml DNase I (Sigma) in IMDM media containing 10% FBS, all incubated at 37°C for 30-40 minutes. To halt collagenase activity, EDTA (5mM) was introduced to all samples. The digested tissues were finally strained through 70μm cell strainers (Falcon) to ensure uniformity in sample preparation.

#### Flow cytometry

Cells prepared for flow cytometric analysis were subjected to a 4-hour incubation with PMA (50 ng/ml) and Ionomycin (500 ng/ml), followed by the addition of Monensin (Biolegend) to the culture for the last 3 hours. The established protocols for surface staining and intracellular cytoplasmic protein staining by eBioscience were diligently followed. LIVE/DEAD Fixable Dead Cell Stain (Thermo Fisher) was employed to stain the single-cell suspensions, which were subsequently fixed using the eBioscience IC fixation buffer. For intracellular cytokine staining, the cells were fixed and permeabilized using eBioscience IC Fixation buffer and 1x permeabilization buffer, respectively. Fluorescent dye-conjugated antibodies were then applied to stain the cells. After a thorough wash with 3% PBS-FCS, the cells were ready for analysis using the Cytek Aurora (5L) flow cytometer. FlowJo v10.2 software facilitated the data analysis.

#### T helper cell culture

Splenic CD4^+^ T helper cells from C57BL/6 mice were purified using the CD4^+^ T Cell Isolation kit (Miltenyi Biotec). 100,000 CD4^+^ T cells were seeded in 200μL on round bottom 96 well tissue culture plate anti-CD3e (2 μg/ml, clone 145-2C11, eBioscience) and anti-CD28 (3 μg/ml, clone 37.51, eBioscience) coated plates in Iscove’s Modified Dulbecco's Medium containing 10% charcoal-stripped serum (without Phenol red), β-mercaptoethanol (55 μM) and recombinant murine IL2 (10 ng/ml, R&D Systems). Charcoal-stripped serum was used to reduce the background of lipid and steroids in the pregnenolone ELISA. To polarise the cells toward T helper 2 (Th2), recombinant murine IL4 (10 ng/ml, R&D Systems), neutralising anti-IFNγ antibody (10 μg/ml, clone XMG1.2, eBioscience) and anti-IL12 antibody (10 μg/ml) were added. The Th2 cells were transferred from the activation plates to resting plates after 72 hours (in a 5% CO2 incubator). The drugs were then added to the Th2 cells with fresh media (serially diluted), and the control wells received DMSO or vehicle. After 48 hours, the plates were centrifuged at 400 X g for 5 min to collect cell supernatant for pregnenolone quantification using ELISA. The cells in each well were used to assess the effect of drug treatment on cell viability.

#### H295R cell culture and pregnenolone ELISA

H295R cells were cultured in a 1:1 mixture of DMEM/Ham’s F-12 nutrient medium with phenol red, supplemented with 2.5% Nu-serum, 1% ITS+ Premix, and 1% penicillin-streptomycin at 37°C in a humidified atmosphere (95% air, 5% CO2).

#### Y1 cell culture

Y1 cells were cultured in Ham’s F-12K medium, supplemented with 2.5% foetal bovine serum, 15% horse serum, and 1% penicillin-streptomycin.

#### Cytotoxicity assay

For the analysis cytotoxicity of the drugs, Th2 cells treated with compounds for 48 hours were stained with a solution composed of 80 μg/ml Acridine Orange (AO) and 40 μg/ml DAPI (Chemometec). The percentage of viable and dead cells were counted using NucleoCounter NC-250 automated cell counter (Chemometec). Percentage viability of cells treated with drugs with respect to the viability of control cells were calculated for identification of cytotoxicity of candidate drugs under study.

#### Quantitative pregnenolone ELISA

*In vitro* differentiated Th2 cells from the 4^th^ day were cultured in the presence of our chosen drugs for 48 hours at a density of 1 × 10^6^ cells per mL in 200μL volume in absence of any further activation. After this period, the plates were spun for 5 minutes at 400 X g at room temperature, and the supernatant was collected for ELISA analysis. H295R and Y1 cells between passages 5 and 10 were seeded in 6-well plates (2 mL medium per well) and allowed to adhere for 24 hours. The cells were then treated with either vehicle (DMSO) or posaconazole at concentrations ranging from 0.78 μM to 25 μM in the IMDM medium without phenol red and supplemented with 10% charcoal-stripped FBS and 1% penicillin-streptomycin. After 48 hours of incubation at 37°C in a humidified atmosphere (95% air, 5% CO2), the culture supernatant was collected for pregnenolone ELISA analysis. Pregnenolone concentration of the collected samples was quantified using the pregnenolone ELISA kit (Abnova) following the manufacturer’s instructions. Absorbance was measured at 450 nm and analysed using GraphPad Prism and Excel.

#### Protein preparation

The available structures of the CYP11A1 were downloaded from PDBe.^30^ Water molecules were deleted and the hydrogen atoms were added before docking using CCDC GOLD suite.^28^ The ligand 22-hydroxy cholesterol and the iron-sulfur clusters present in the crystal structure removed before docking. The Heme cofactor within the active site of the CYP11A1 contributes to the ligand bindings, and therefore it was retained during protein preparation for virtual screening. *In silico* mutagenesis of the protein was done using PyMol.

#### Ligand preparation

Three libraries of compounds were downloaded from the ZINC15 database^27^ (https://zinc15.docking.org), namely the FDA subset, World subset and Investigational only subset. A KNIME workflow (Figure S4) using RdKit nodes (RDKit: Open-source cheminformatics (https://www.rdkit.org) were used to generate 3D conformers of the ligands. Missing hydrogen atoms were added prior to the conversion. Finally, CCDC conformer generator was used to make a single conformer of each ligand.

#### Virtual screening

A brief overview of the screening methodology is represented in Figure 1A. CCDC GOLD suite was used for the virtual screening. The prepared structure of the target protein was used to perform a preliminary docking experiment using GOLD, where the crystallised ligand or Cholesterol was redocked with the protein. Consensus scoring was used to determine the best scoring function to be used for further docking runs. CHEMPLP provided the best performance. This docking configuration was used to perform virtual screening with all three libraries of the compounds.

Selection of compounds for experimental validation: The docking results of all three subset libraries were combined, and the top 100 poses with the highest docking scores were shortlisted for further analysis. DataWarrior^29^ was used to cluster the 100 compounds with similarity cut-off criteria 0.8. The top-scored one was taken for further analysis in the clusters with more than one compound. A total of 69 compounds were taken for visual analysis after clustering. PyMol and Discovery studio visualizer were used for visual analysis and further evaluation of the docking poses. CCDC Mogul^31^^,^^32^ was used to assess ligand geometries.

### Quantification and statistical analysis

The statistical analysis was performed using GraphPad Prism Software. The data were analysed by either one-way or two-way ANOVA. The Dunnet multiple comparison test was employed for one-way ANOVA, and for two-way ANOVA, Sidak multiple comparison test was used (Figure 1). Unpaired two-tailed t-test has also been applied in some experiments (Figure 3). The *p* values < 0.05 were considered significant. The values were presented as mean ± standard deviation (SD) or standard error of mean (SEM) as mentioned in the respective figure legends. The exact value of N and the representation of N are explicitly mentioned in the figure legends.
