# Supplementary material for: Drug repurposing reveals posaconazole as a CYP11A1 inhibitor enhancing anti-tumor immunity
Source: iScience. 2025 Apr 18;28(5):112488. doi: 10.1016/j.isci.2025.112488 (PMC12124671; doi:10.1016/j.isci.2025.112488)
Supplement: Document S1. Figures S1–S4 and Tables S1 and S2 [file mmc1.pdf]

iScience, Volume *28*

Supplemental information

Drug repurposing reveals posaconazole as a CYP11A1 inhibitor enhancing anti-tumor immunity

Jhuma Pramanik, Sanu Korumadathil Shaji, Megan Zaman, Bethany Brown, Baojie Zhang, Yumi Yamashita-Kanemaru, Natalie Z.M. Homer, Hosni A.M. Hussein, Qiuchen Zhao, Klaus Okkenhaug, Rahul Roychoudhuri, Abhik Mukhopadhyay, and Bidesh Mahata

Figure S1

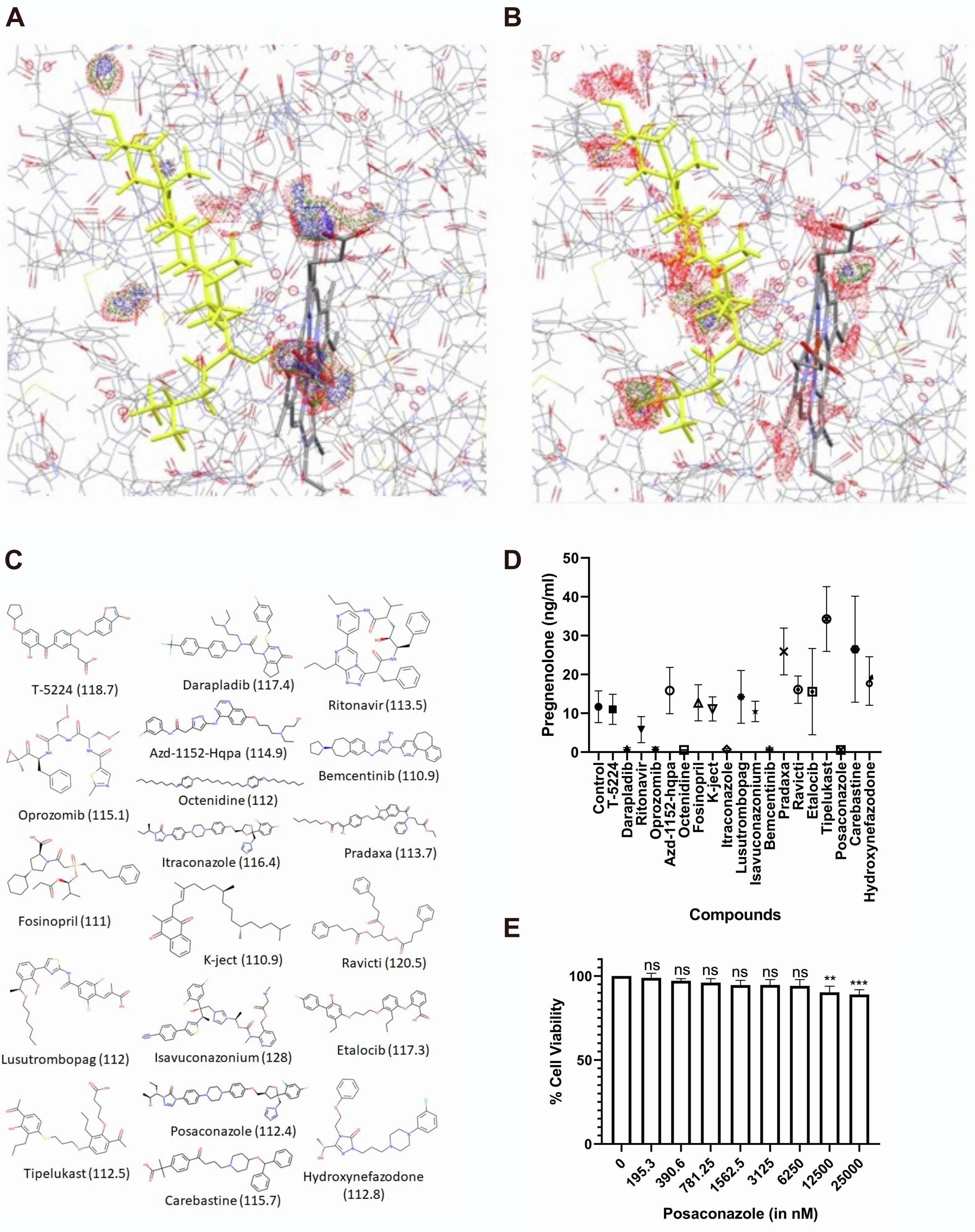
A
B
C
T-5224 (118.7)
Darapladib (117.4)
Ritonavir (113.5)
Oprozomib (115.1)
Azd-1152-Hqpa (114.9)
Bemcentinib (110.9)
Octenidine (112)
Itraconazole (116.4)
Pradaxa (113.7)
Fosinopril (111)
K-ject (110.9)
Ravicti (120.5)
Lusutrombopag (112)
Isavuconazonium (128)
Etalocib (117.3)
Tipelukast (112.5)
Posaconazole (112.4)
Hydroxynefazodone (112.8)
Carebastine (115.7)
D
Pregnenolone (ng/ml)
50
40
30
20
10
0
Control
T-5224
Darapladib
Ritonavir
Oprozomib
Azd-1152-hqpa
Octenidine
Fosinopril
K-ject
Itraconazole
Lusutrombopag
Isavuconazonium
Bemcentinib
Pradaxa
Ravicti
Etalocib
Tipelukast
Posaconazole
Carebastine
Hydroxynefazodone
Compounds
E
% Cell Viability
100
50
0
ns
ns
ns
ns
ns
ns
**
***
0
195.3
390.6
781.25
1562.5
3125
6250
12500
25000
Posaconazole (in nM)

Figure S1. In silico screening for the identification of potential CYP11A1 inhibitors. Related to Figure 1.

A. Superstar Propensity map of CYP11A ligand binding region. Propensity of 2.0 is coloured red, 4.0 is coloured green and 8.0 is coloured blue. Propensity map generated using the Carbonyl Oxygen Probe. The carbonyl oxygen can accept hydrogen bonds, which enable to identify the region in the binding pocket favouring hydrogen acceptor group in the ligand molecule.

B. Propensity map generated using the Aliphatic CH Carbon Probe. Aliphatic CH Carbon Probe is useful for the detection of regions in the binding cavity favouring hydrophobic group in the ligand molecule.

C. Chemical structures and GOLD score of top hits identified after virtual screening and visual observation.

D. In vitro validation of CYP11A1 inhibitors. A. Screening for CYP11A1 inhibitors (top hits) for their inhibitory property. In vitro generated Th2 cells were treated with 10uM of the drug for 48 hours and pregnenolone concentration was measured by ELISA. B. Posaconazole does not induce cytotoxicity in mice Th 2 cells. Cell viability was assessed using AO/DAPI staining in an automated cell counter. Percentage cell viability was calculated based on the viability of control cells treated with DMSO. (N=3,One-Way ANOVA with Dunnet multiple comparison, *$P \leq 0.05$, **$P \leq 0.01$, ***$P \leq 0.001$)

Figure S2

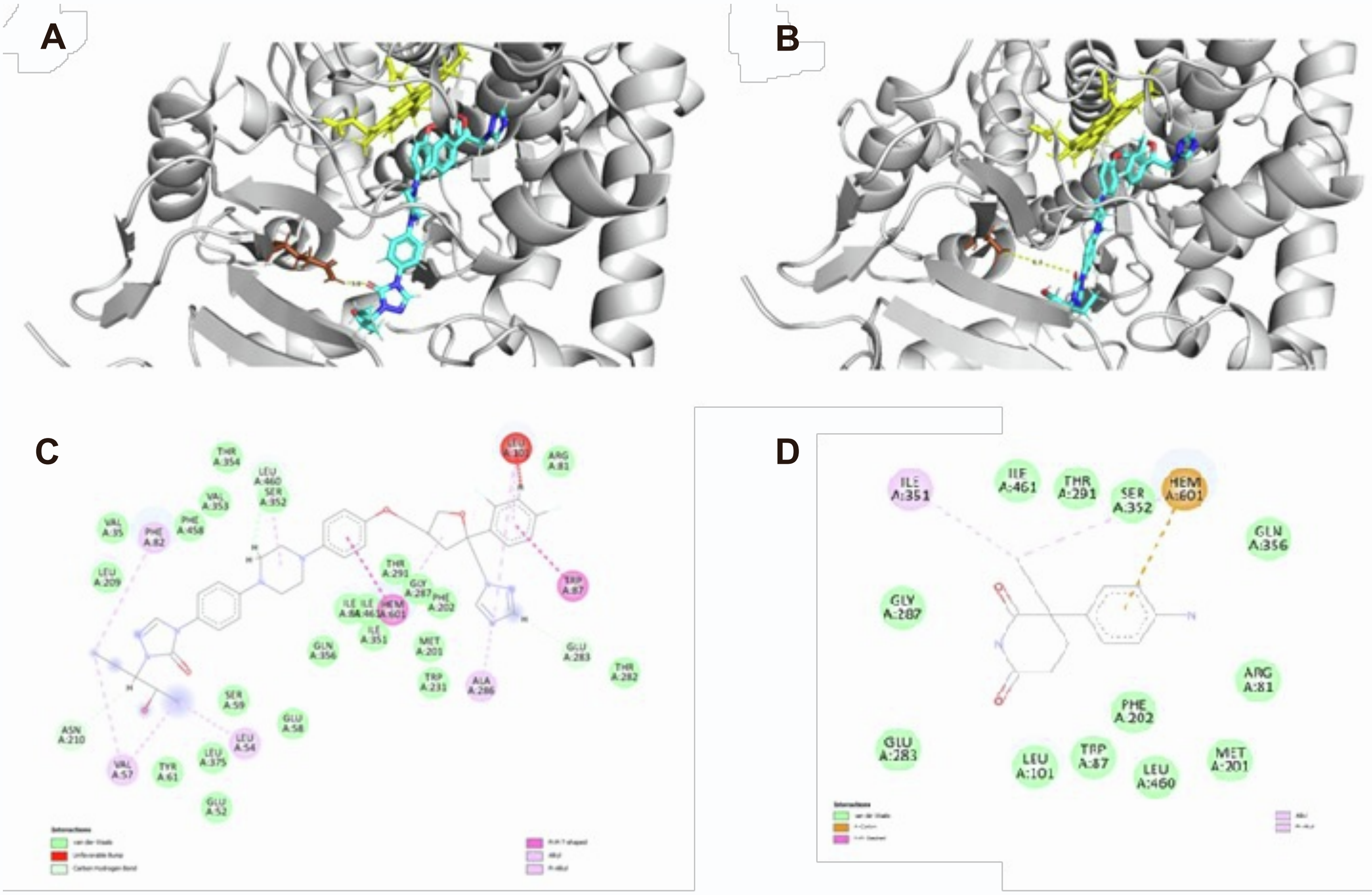

Figure S2. Analysis of docking poses**.** Related to Figure 2.

A. Mutation of Gln377 to alanine reduces binding stability of posaconazole. Posaconazole in CYP11A1 binding pocket forming hydrogen bonds between oxygen in the azole group and Gln377.

B. Posaconazole in mutated CYP11A1 binding pocket. Mutation of Gln377 to Ala increases the distance between oxygen in the azole group and CYP11A1 from 3 Å to 6.7 Å, resulting in the loss of hydrogen bond formation.

C. 2D diagram of docking pose of Gln377 to Alanine mutated CYP11A1 and posaconazole.

D. 2D diagram of docking pose of CYP11A1 and aminoglutethimide (GOLD score = 60).

Figure S3

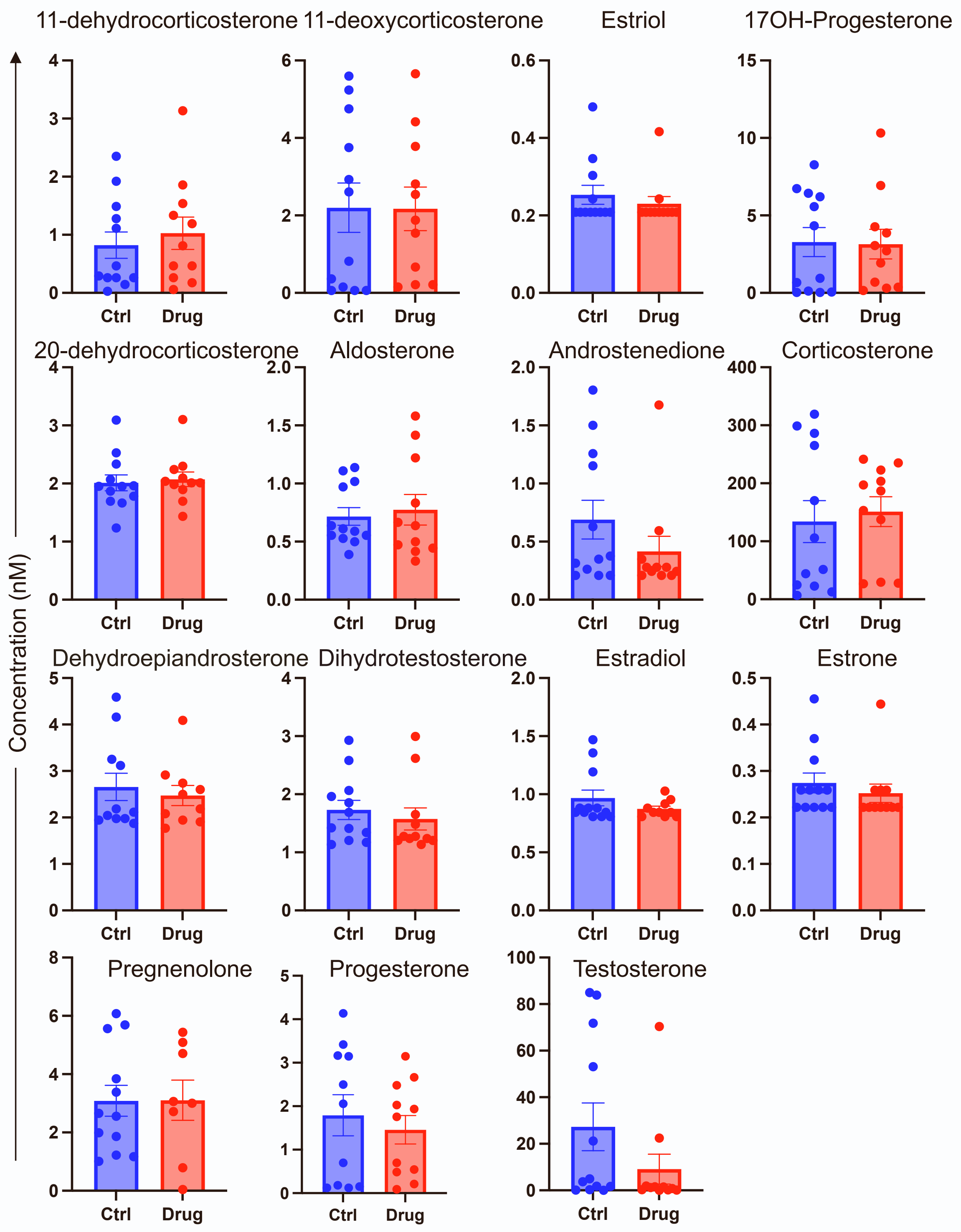

Figure S3. Systemic steroid profiling of posaconazole-treated and vehicle-treated mice by measuring serum steroid concentrations using LC-MS/MS. Related to Figure 3.

Figure S4

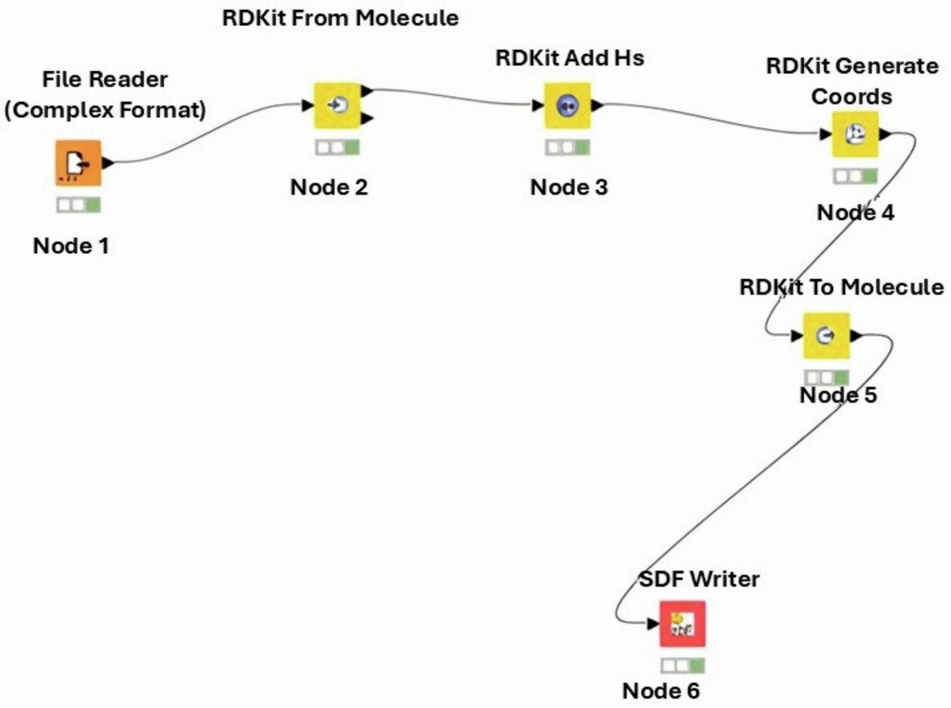

Figure S4. KNIME pipeline used to convert SMILES to 3D structure of compounds for virtual screening. Related to STAR Methods.

Table S1: R_{free}, resolution and R Factor values of CYP11A1 structures. Related to Figure 1.

PDB Accession Code	R factor	R_{free}	Resolution (Å)
**3NA0**	**0.203**	**0.245**	**2.5**
**3N9Y**	**0.207**	**0.242**	**2.1**
**3NA1**	**0.195**	**0.232**	**2.25**
**3N9Z**	**0.211**	**0.243**	**2.17**

Table S2: Top 100 compounds after GOLD docking with GOLD PLP fitness score and cluster number. Clustering was performed using DataWarrior. Related to Figure 1.

ZINC ID	Gold.PLP.Fitness	Cluster No
ZINC000003920355	111.053	1
ZINC000003831151	122.6484	2
ZINC000003831151	120.5102	2
ZINC000003920372	117.3718	3
ZINC000004097344	116.4883	3
ZINC000004097344	116.0798	3
ZINC000072131401	115.7787	3
ZINC000060392779	114.6387	3
ZINC000003830976	113.6441	3
ZINC000253387884	113.1819	3
ZINC000028639340	112.4058	3
ZINC000028007119	111.8825	3
ZINC000004097343	111.8503	3
ZINC000150339331	111.6247	3
ZINC000028639340	111.4614	3
ZINC000150339328	111.4494	3
ZINC000003830974	111.0444	3
ZINC000038945666	120.5159	4
ZINC000038945666	118.7188	4
ZINC000049637509	127.9932	5
ZINC000029571072	120.4728	5
ZINC000049637509	118.0012	5
ZINC000060392785	133.5119	6
ZINC000028108825	122.9271	6
ZINC000060392785	114.7394	6
ZINC000100016058	110.6859	7
ZINC000008035268	116.4525	8
ZINC000008035268	113.6131	8
ZINC000161527403	112.9287	9
ZINC000161527507	112.825	9
ZINC000043202141	115.1058	10
ZINC000001914117	111.9685	11
ZINC000013888387	111.2552	11
ZINC000004632133	110.9041	12

ZINC000255991347	122.7362	13
ZINC000058485982	112.9522	13
ZINC000065731166	114.2049	14
ZINC000100052151	118.0854	15
ZINC000150376965	113.6916	15
ZINC000003930411	112.1246	16
ZINC000034775091	114.0194	17
ZINC000003938642	112.0949	18
ZINC000003776894	110.9333	18
ZINC000028711707	113.2602	19
ZINC000100499682	121.2848	20
ZINC000138250800	116.9125	20
ZINC000003796820	112.4836	21
ZINC000206181179	110.6997	22
ZINC000030160243	119.5812	23
ZINC000049833385	118.3413	23
ZINC000059676426	113.3432	24
ZINC000043175204	130.1448	25
ZINC000003914388	121.5953	26
ZINC000118713970	115.1198	27
ZINC000049756486	112.5486	28
ZINC000014261842	111.0055	29
ZINC000008214632	112.0465	30
ZINC000072190222	111.751	31
ZINC000139805602	120.3976	32
ZINC000196068306	116.5722	33
ZINC000003944422	113.5104	33
ZINC000195932465	119.3551	34
ZINC000016697102	114.9227	35
ZINC000169734819	112.5539	36
ZINC000008214358	117.8934	37
ZINC000003930629	117.1421	38
ZINC000003842798	117.3522	39
ZINC000051951669	110.8591	40
ZINC000029238439	113.3398	41
ZINC000049756387	123.4553	42
ZINC000036330812	112.9789	43
ZINC000003961398	115.6058	44
ZINC000003816510	118.7377	45
ZINC000008214391	115.713	46
ZINC000072190199	117.4642	47
ZINC000139868161	116.5809	47
ZINC000003777825	113.2677	48
ZINC000014300189	110.8227	49
ZINC000003935633	113.3156	50
ZINC000113459996	113.8024	51

ZINC000004215193	115.6764	52
ZINC000084759273	111.9515	53
ZINC000003831329	110.863	54
ZINC000095598449	116.7526	55
ZINC000003806063	110.7195	56
ZINC000001566899	113.4403	57
ZINC000008552165	112.8592	58
ZINC000215581115	110.9871	58
ZINC000003943279	113.6837	59
ZINC000031476880	113.8545	60
ZINC000060292466	114.8039	61
ZINC000053073961	114.0516	62
ZINC000003875376	116.9431	63
ZINC000003945332	118.7529	64
ZINC000008551087	112.4772	65
ZINC000021981274	112.8417	66
ZINC000021981272	111.9019	66
ZINC000095604030	116.0229	67
ZINC000033956090	110.8082	68
ZINC000033972993	111.5648	69
